# Pilot Study Assessing the Impact of Intrathecal Administration of Variants AAV-PHP.B and AAV-PHP.eB on Brain Transduction in Adult Rhesus Macaques

**DOI:** 10.3389/fbioe.2021.762209

**Published:** 2021-11-15

**Authors:** Marie-Laure Arotcarena, Sandra Dovero, Nathalie Biendon, Nathalie Dutheil, Vincent Planche, Erwan Bezard, Benjamin Dehay

**Affiliations:** ^1^ CNRS, IMN, UMR 5293, Univ. Bordeaux, Bordeaux, France; ^2^ Centre Memoire de Ressources et de Recherches, Pôle de Neurosciences Cliniques, CHU de Bordeaux, Bordeaux, France

**Keywords:** adeno-associated virus, intrathecal injection, CNS, non-human primate, animal model

## Abstract

Adeno-associated virus (AAV) vectors are increasingly used as an effective and safe approach to deliver genetic material to the central nervous system (CNS). The AAV9-derived variants, AAV-PHP. B and AAV-PHP.eB, reportedly broadly transduce cells throughout the CNS compared to the original serotype 9, AAV9. As non-human primate data are scarce, we here evaluated the CNS transduction efficiencies after lumbar intrathecal bolus delivery of identical doses of either AAV-PHP. B:CAG-EGFP or AAV-PHP. eB:CAG-EGFP in rhesus macaque monkeys. AAV-PHP.eB achieved a more efficient and widespread CNS transduction compared to AAV-PHP.B. We report a strong neuronal and oligodendroglial tropism for both variants in the putamen and in the hippocampus. This proof-of-concept experiment highlights the potential value of intrathecal infusions of AAV-PHP.eB to distribute genetic material in the CNS with cell-type specificity and introduces a new opportunity to model brain diseases in rhesus macaque monkeys and further develop gene therapies targeting the CNS in humans.

## Introduction

The use of non-pathogenic adeno-associated virus (AAV) vectors has emerged as an effective and safe approach for both preclinical modeling and therapeutic methods for neurological disorders (Bourdenx et al., 2014; Deverman et al., 2018; Hudry and Vandenberghe, 2019; Nectow and Nestler, 2020; Fajardo-Serrano et al., 2021). In particular, specific serotypes of AAV, such as recombinant adeno-associated virus serotype 9 (AAV9) and pseudotype rhesus-10 (AAVrh.10), have shown unique properties to target the central nervous system (CNS) in comparison to most AAV serotypes (Foust et al., 2009). Since both serotypes share the ability to cross the blood-brain barrier (BBB) in newborns, they may be delivered through various administration routes (Bourdenx et al., 2014; Bedbrook et al., 2018). They are associated with widespread, long-term transduction in nondividing cells in rodents (Cearley and Wolfe, 2006; Duque et al., 2009; Foust et al., 2009; Zhang et al., 2011; Chansel-Debordeaux et al., 2017; Chansel-Debordeaux et al., 2018) and non-human primates (NHPs) (Dehay et al., 2012; Gray et al., 2013; Samaranch et al., 2013; Hinderer et al., 2014; Yang et al., 2014; Bey et al., 2020), making them a powerful tool for delivering genetic material to the CNS. Recently, a novel AAV9-derived variant that far more efficiently crosses the mouse BBB (named AAV-PHP. B) has been identified by screening a library of AAV9 vectors carrying random mutations at the capsid surface (Deverman et al., 2016). AAV-PHP. B transfers genetic information throughout the CNS with high efficiency compared to the gold-standard AAV9 (Deverman et al., 2016) and transduces most brain cells (approximately 50–100% of neurons and 80% of astrocytes) across multiple CNS regions after peripheral delivery in mice (Deverman et al., 2016; Allen et al., 2017; Gao et al., 2017; Morabito et al., 2017; Hordeaux et al., 2018; Rincon et al., 2018) and rats (Jackson et al., 2016). However, at odds with rodent data, intravenously delivered AAV-PHP. B did not demonstrate the same capabilities in both marmosets (6 weeks survival) (Matsuzaki et al., 2018) and rhesus macaques (3 weeks survival) (Hordeaux et al., 2018).

Pursuing the effort towards identification of AAVs with tremendous transduction potential, the group that discovered AAV-PHP. B recently reported an enhanced AAV-PHP. B variant (AAV-PHP.eB) that shows further improvements in neurons and glia transduction efficiency throughout the CNS after intravenous delivery in adult mice (Chan et al., 2017; Mathiesen et al., 2020), or after intravascular (Dayton et al., 2018) and cisterna magna (Chatterjee et al., 2021) administration in adult rats. As a face-to-face comparison of AAV-PHP. B and AAV-PHP. eB is currently missing in NHP, we here compared the CNS transduction efficacy of these two new variants after intrathecal injection of the same dose in the rhesus macaque (*Macaca mulatta*). Using the EGFP reporter transgene to characterize viral biodistribution, we observe enhanced AAV-PHP. eB-mediated viral transduction compared to AAV-PHP. B throughout the CNS. Furthermore, we report a neuronal and oligodendroglial tropism for both variants in the NHPs brain.

## Materials and Methods

### Animals

Two female adult rhesus monkeys (*Macaca mulatta*), weighing ∼6–7 kg (9–10 years old), were used in this study. The monkeys were housed in the animal facility of the Institute of Neurodegenerative Diseases (UMR CNRS 5293) under standard conditions (12/12 h day/night cycle, ∼60% humidity, and ∼22°C). All experimental protocols comply with the Council Directive of 2010 (2010/63/EU) of the European Community and the National Institute of Health Guide for the Care and Use of Laboratory Animals. The proposed research has received French Ethical Committee for Animal Research CE50 (agreement number: 12286).

### Viral Vectors and Surgical Procedures

#### Production of the Viral Vector

Recombinant AAV vectors (rAAV) were produced at the vector core facility of our lab. rAAV were generated by polyethylenimine mediated triple transfection of low passage HEK-293T/17 cells (ATCC; cat number CRL-11268). The AAV expression plasmid pAAV2-CAG-EGFP-WPRE-pA (enhanced green fluorescent protein (EGFP) under the control of cytomegalovirus enhancer fused to the chicken beta-actin (CAG) promoter) was co-transfected with plasmids encoding the AAV2 rep and AAV-PHP. B or AAV-PHP. eB cap genes (pUCmini-iCAP-PHP.B, Addgene # 103,002; pUCmini-iCAP-PHP.eB, Addgene # 103005) and adenoviral helper functions (pAdΔF6, Addgene # 112867) (Xiao et al., 1998). AAV vectors were purified as previously described (Zolotukhin et al., 2002). Cells were harvested 72 h post-transfection, resuspended in lysis buffer (150 mM NaCl, 50 mM Tris-HCl pH 8.5), and lysed by three freeze-thaw cycles (37°C/−80°C). The cell lysate was treated with 150 units/ ml Benzonase (Sigma Aldrich, St Louis, MO, United States) for 1 h at 37°C, and the crude lysate was clarified by centrifugation. Vectors were purified by iodixanol step gradient centrifugation and concentrated and buffer-exchanged into Lactated Ringer’s solution (Baxter, Deerfield, IL, United States) using vivaspin20 100 kDa cut off concentrator (Sartorius Stedim, Goettingen, Germany). Titrations were performed at the platform study of the transcriptome (Neurocentre Magendie, INSERM U862, Bordeaux, France). The genome-containing particle (gcp) titer was determined by quantitative real-time PCR using the Light Cycler 480 SYBR green master mix (Roche, cat # 04887352001) with primers specific for the AAV2 ITRs (fwd 5′-GGA​ACC​CCT​AGT​GAT​GGA​GTT-3′; rev 5′-CGG​CCT​CAG​TGA​GCG​A-3′) (Aurnhammer et al., 2012) on a Light Cycler 480 instrument. Purity assessment of vector stocks was estimated by loading 10 µL of vector stock on 10% SDS acrylamide gels. According to the manufacturer’s instructions, total proteins were visualized using the Krypton Infrared Protein Stain (Life Technologies). The production and the titration for both AAV9-derived variants have been made in parallel to avoid any bias.

#### Intrathecal Injections

Injections were performed under aseptic conditions and isoflurane and O_2_ deep anaesthesia, following induction with ketamine (10 mg/  IM) and atropine (0.05 mg/ kg IM). Body temperature, heart rate, blood pressure, and SpO_2_ were monitored throughout all procedures. The injection site has been prepared by initially wiping the area with sponges soaked in 70% isopropyl alcohol and/or betadine scrub, allowed to dry. The animal was placed in lateral recumbence, and a 22G cannula for lumbar puncture was introduced into the L4-L5 intrathecal space. The presence of CSF in the cannula after the removal of the stylet certified the intrathecal placement. A solution containing AAV-PHP. B:CAG-EGFP-WPRE-pA was then injected at 1.0 × 10^12^ vg for one monkey, corresponding to 180 µL. A solution containing AAV-PHP. eB:CAG-EGFP-WPRE-pA was injected at 1.0 × 10^12^ vg per monkey, corresponding to 100 µL. The intrathecal administration was followed by a flush of 0.4 ml of sterile saline. The cannula was removed, and the animal was maintained in lateral recumbence for 1 hour until recovery from anaesthesia. Animals were longitudinally followed and euthanized 2 months post-injection.

#### Post-Mortem Processing

Animals were deeply anesthetized with a lethal dose of pentobarbital (50 mg/kg) and transcardially perfused with room-temperature 0.9% saline solution (containing 1% heparin) following accepted European Veterinary Medical Association guidelines. Brains, spinal cords, and livers were removed quickly after death. Each brain was then dissected along the midline, and each hemisphere was divided into three parts. Each spinal cord was divided into three parts (cervical thoracic, lumbar). These tissues were postfixed in a large volume of 4% buffered paraformaldehyde solution for 1 week at 4°C and cryoprotected in successive baths of 20–30% sucrose solution diluted in 0.1 M phosphate buffered saline (PBS) at 4°C until they sunk. Finally, brains were frozen by immersion in an isopentane bath at −55°C for 5 min and stored immediately at −80°C until sectioning. No samples were excluded from analysis in these studies.

### Immunohistochemical Analysis

#### Transgene Expression

Brains, spinal cords and livers were sectioned in 50 μm thick serial free-floating coronal sections in a Leica CM3050S cryostat (Leica Microsystems, Wetzlar, Germany) at −20°C, collected in PBS Azide 0.2% and store at 4°C until they were processed for EGFP immunoreactivity. Coronal free-floating sections were incubated with a mouse monoclonal antibody raised against GFP (Invitrogen Anti-GFP Monoclonal (3E6), Catalog #A-11120; 1:1000) for one night at room temperature and revealed by an anti-mouse peroxidase EnVision™ system (DAKO, K4011) followed by DAB incubation. Free-floating sections were then mounted on gelatinized slides, counterstained with cresyl violet, dehydrated, and coverslipped before further analysis. Sections were scanned in a high-resolution scanner (PanScan, 3D Histech) at ×20 magnification, and the quantification of GFP-positive signal was estimated by an immunostaining-positive surface quantification in the different brain regions with the Mercator software (Explora Nova). All analyses were performed blinded to the researcher.

#### Immunofluorescence Analysis

To assess brain cellular subtype markers (i.e., neurons, microglia, astrocytes, and oligodendrocytes) co-expressed with EGFP, striatal and hippocampal monkey sections were incubated simultaneously with two antibodies: a chicken polyclonal antibody against GFP (Abcam, ab13970, 1:1000) and, respectively, a mouse monoclonal antibody against NeuN (Millipore, Darmstadt, Germany, MAB377, 1:1000) or a rabbit monoclonal against human Iba1 (FUJIFILM Wako Pure Chemical Corporation, 019-19741, 1:1000) or a mix of mouse monoclonal antibodies against GFAP and S-100 (Sigma-Aldrich (clone GA5), 1:2000/Abcam, ab7852, 1:1000) or a mouse monoclonal antibody against CNPase (Abcam, ab237961, 1:1000) for one night at room temperature. Incubation with secondary antibodies was done sequentially with a goat anti-chicken AlexaFuor 488 (Abcam, A11039, 1:400) and a goat anti-mouse AlexaFluor 568 (Invitrogen, A11031, 1:400) or a goat anti-rabbit AlexaFluor 568 (Invitrogen, A11036, 1:400) 1h30 at room temperature. Sections were mounted on non-gelatinized slides and coverslipped using fluorescent mounting media without DAPI (Vector Labs). Images were acquired using a Zeiss SP5 confocal microscope.

#### Quantitative Analysis

For each NHP, one slice of the median part of the striatal or the hippocampal level was selected for each cell-type-specific marker/EGFP double-labeling immunofluorescence. Ten to thirteen images were captured at ×40 magnification with a confocal laser microscope (LSM780; Zeiss). Cellular tropism is reported as a percentage of total EGFP-positive or EGFP-negative cells per cell type counted in all images acquired [corresponding to a total of NeuN-positive (92–119 cells in the striatum, 116–124 cells in the hippocampus), Iba1-positive (33–45 cells in the striatum, 36–43 cells in the hippocampus), GFAP/S-100-positive (33–47 cells in the striatum, 84–83 cells in the hippocampus), CNPase-positive (101–111 cells in the striatum, 32–71 cells in the hippocampus) cells]. The percentage corresponds to the proportion of EGFP-positive or EGFP-negative cell divided by the total number of counted cells, calculated independently for each cell type. For example, for the percentage of transduced neurons, we calculated the number of EGFP-positive neurons divided by the total number of analysed neurons expressed in percentage.

### Biochemical Analysis

#### Protein Extraction

We used 2 mm^3^ formalin-fixed tissue per animal. Tissue patches were extracted using the Qproteome FFPE Tissue Kit (Qiagen), and then quantified using the Lowry method (Biorad RC DC Protein Assay Kit) following the manufacturer’s instructions. Based on total protein concentrations calculated from the assay, aliquots of tissue lysates corresponding to 10 μg were prepared for each structure in Laemmli buffer (Tris-HCl 25 mM pH = 6.8, glycerol 7.5%, SDS 1%, DTT 250 mM and bromophenol blue 0.05%). Samples were loaded into 12% SDS-PAGE gel, followed by electrophoretic transfer onto 0.2 l m nitrocellulose membranes (Bio-Rad, Hercules, CA, United States). Membranes were blocked in phosphate buffer saline (PBS) with 5% dry skimmed milk and probed with antibodies against GFP (JL-8; Clontech, Mountain View, CA, Living Colors, 1:1000) overnight. Anti-actin (1:5,000; Sigma) was used to control equal loading. Appropriate secondary antibodies coupled to peroxidase were revealed using a Super Signal West Pico Chemiluminescent kit (Immobilon Western, Chemiluminescent HRP substrate, Millipore) and analysed with Image Lab software 5.0 (Bio-Rad, Hercules, United States).

## Results

### AAV-PHP.eB:CAG-EGFP Displayed Greater GFP Transduction Efficiency

**FIGURE 1:**
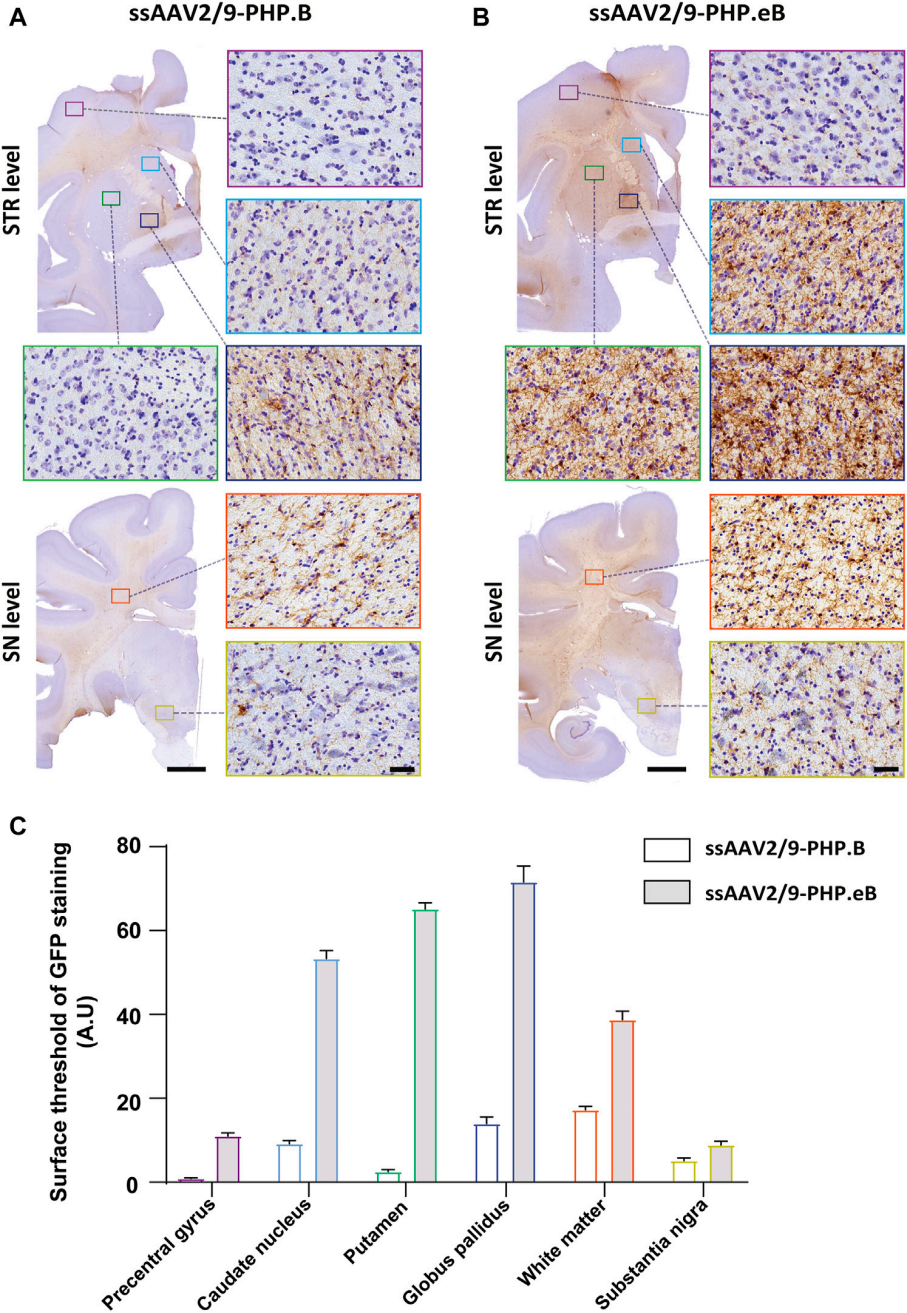
Stronger widespread brain EGFP distribution after intrathecal injection of AAV-PHP. eB compared to variants AAV-PHP. B in non-human primates. **(A–B)** Representative coronal brain sections at two different rostrocaudal levels (top, “STR” = striatal level, AC0; bottom, “SN” = Substantia Nigra level, AC-8) of EGFP immunostaining (left) and illustrative photomicrographs (right) of EGFP immunostaining in the precentral gyrus (purple square), in the caudate nucleus (light blue square), in the putamen (green square), in the globus pallidus (dark blue square), in the white matter (orange square) and the substantia nigra (yellow square) in monkeys injected with either AAV-PHP. B **(A)** or AAV-PHP. eB **(B)**. Scale bars = 5 mm (left, sections) and 40 µm (right, insets). **(C)** Quantitative analysis of GFP immunostaining measured by the surface threshold in all analyzed regions, including the precentral gyrus, the caudate nucleus, the putamen, the globus pallidus, the white matter, and the substantia nigra in AAV-injected monkeys. Data represent mean ± SEM.

**FIGURE 2:**
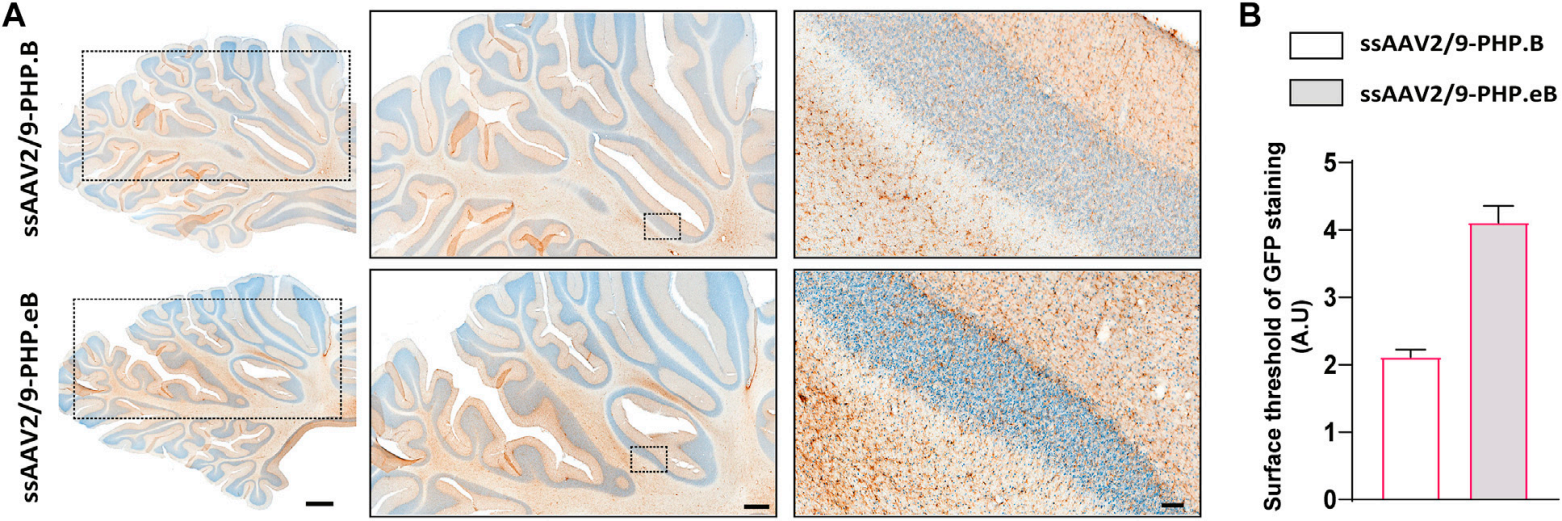
Stronger widespread brain EGFP distribution after intrathecal injection of AAV-PHP. eB compared to variants AAV-PHP. B in the cerebellum of non-human primates. **(A)** Representative coronal brain sections at the cerebellum level (AC-17 mm) of EGFP immunostaining (left) and illustrative photomicrographs (middle and right) of EGFP immunostaining in the cerebellum in monkeys injected with either AAV-PHP. B (up) or AAV-PHP. eB (down). Scale bars = 2 mm (left, sections), 1 mm (middle, sections) and 100 µm (right, insets). **(B)** Quantitative analysis of EGFP immunostaining measured by surface threshold analyzed on four sections of the cerebellum in AAV-injected monkeys. Data represent mean ± SEM.

**FIGURE 3:**
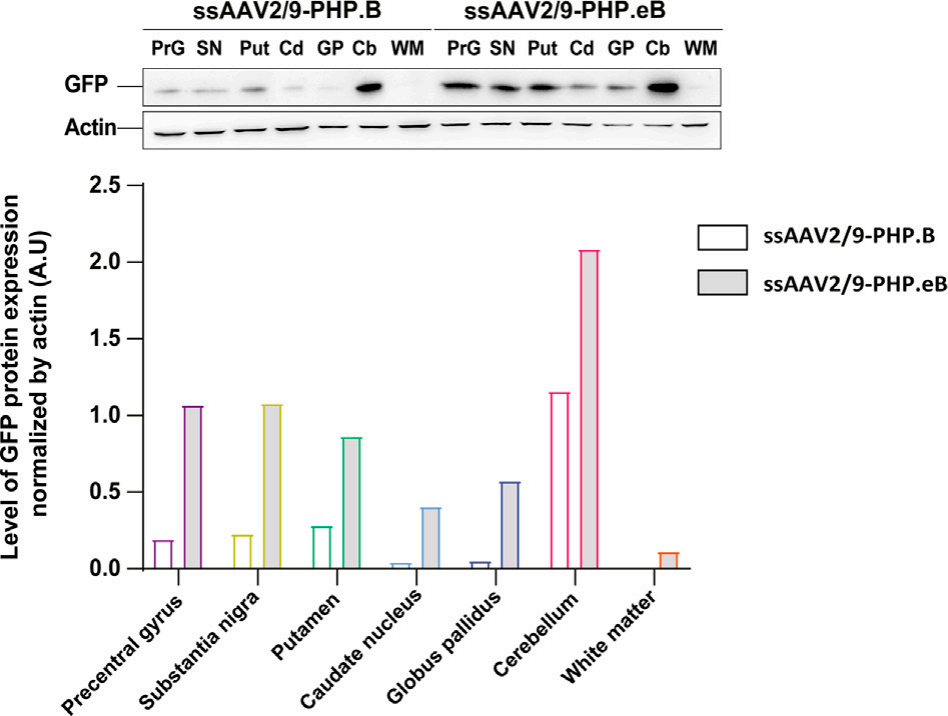
Stronger widespread brain EGFP distribution after intrathecal injection of AAV-PHP. eB compared to variants AAV-PHP.B. Representative images and quantification of EGFP expression analyzed across brain lysates of AAV-injected monkeys.

To confirm the ability of AAV9-derived variants, AAV-PHP. B and AAV-PHP. eB, to transduce neural cells after intrathecal delivery, we proceeded with a pilot study in NHPs (Table 1). Naïve 9-to 10-year-old adult rhesus macaques (*n* = 1 per vector) received single intrathecal injections of viruses at a dose of 1.0 × 10^12^ vector genomes (vg) per animal (1.5 × 10^11^ vg/ kg body weight), a quantity that was comparable with the highest dose used in the mouse studies (Chan et al., 2017). Both NHPs tolerated the vectors well. Two months later, both monkeys were euthanized. We first qualitatively evaluated the relative transduction profiles of AAV-PHP. B:CAG-EGFP (Figure 1A) and AAV-PHP. eB:CAG-EGFP (Figure 1B). The monkey injected with AAV-PHP. eB:CAG-EGFP showed an extensive CNS transduction and a more intense expression of EGFP throughout the brain than the monkey injected with AAV-PHP. B:CAG-EGFP. A quantitative unbiased evaluation of the EGFP-positive cell surface staining confirmed these observations (Figure 1C). Several cortical and subcortical structures such as the precentral gyrus, the putamen, the caudate nucleus, the globus pallidus, the white matter, and the substantia nigra showed EGFP staining suggesting transduction of these structures by both variants. Deeper brain structures (such as the putamen, the caudate nucleus and the globus pallidus) were even more EGFP-positive in AAV-PHP. eB-injected monkey. Similarly, a broad viral transduction was observed in the cerebellum for both variants with stronger EGFP-positive staining observed for the AAV-PHP. eB-injected monkeys (Figures 2A,B). Immunoblots of the same brain areas showed significantly higher levels of EGFP expression in the AAV. PHP.eB-injected monkey compared to that of AAV.PHP.B-injected monkey (Figure 3).

**TABLE 1:** Summary of study subjects.

Animal #	Virus	Sex	Age (year)	Weight at injection (kg)	Viral titers (vg/ml)	Volume (µL)	Dose (vg/kg)	Sacrifice (days after injection)
1	PHP.B	female	9.6	6,5	5.33 × 10^12^	180	1.5 × 10^11^	60
2	PHP.eB	female	9.7	6,7	1.04 × 10^13^	100	1.5 × 10^11^	60

### Both Vectors Achieved a Neuronal and Oligodendroglial Tropism in the Putamen

**FIGURE 4:**
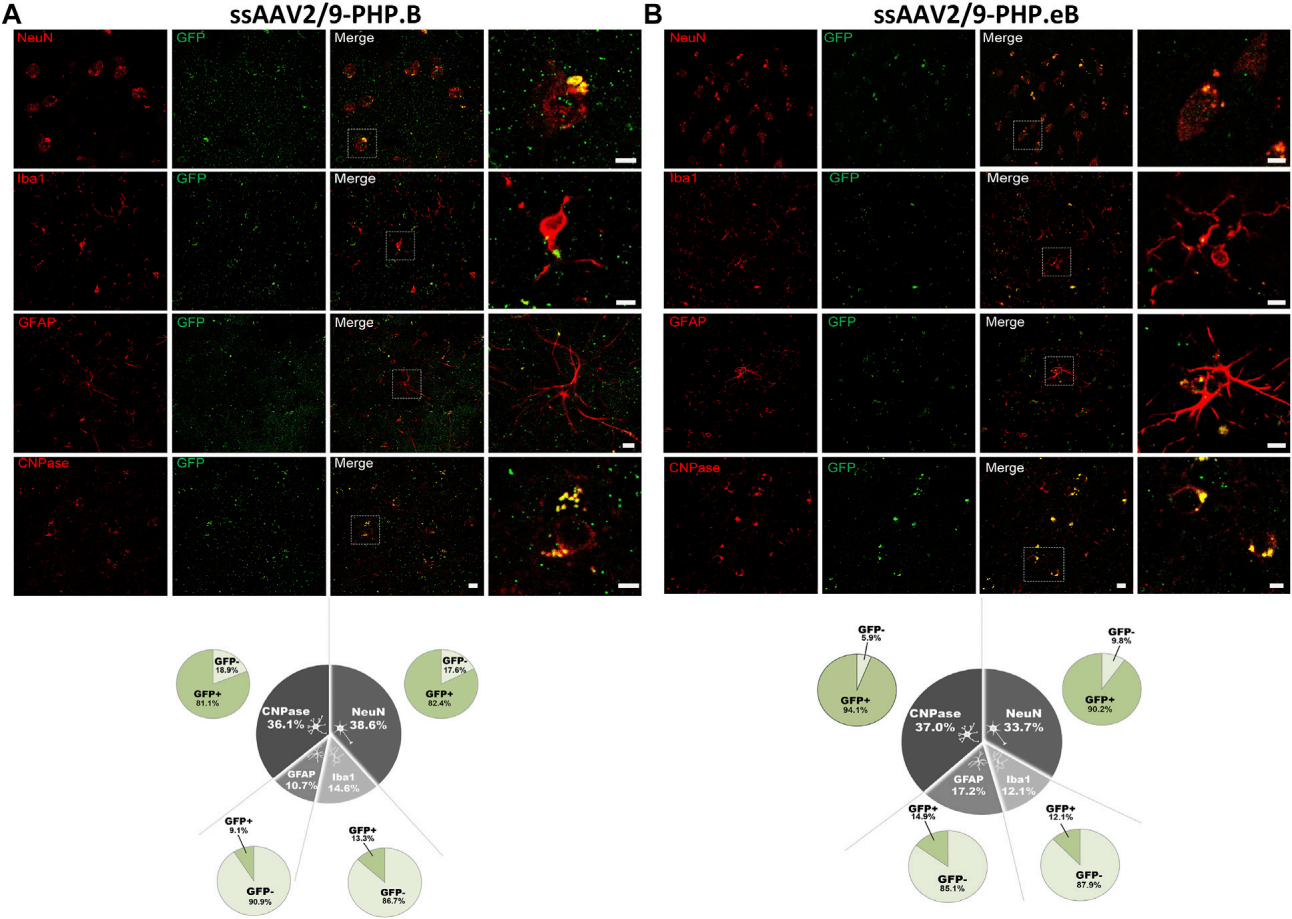
Neuronal cell-type specificity resulting from AAV-PHP. B and AAV-PHP. eB injections in the putamen of non-human primates. **(A–B)** Representative images (top) and quantifications (down) of striatal sections from AAV-PHP. B-injected monkey **(A)** and AAV-PHP. eB-injected monkey **(B)** immunofluorescently labeled with anti-GFP antibody (green) and either a neuronal (NeuN), microglia (Iba1), astrocytic (GFAP + S-100) or oligodendroglial (CNPase) marker (red). Merging of the signals produced colocalization, thus confirming cellular transduction. Scale bar: 20 µm; 10 µm (high magnification). Quantitative analysis of percentage of cell types counted for each viral vector in the putamen of injected monkeys (grey) and percentage of cells transduced (dark green) or not (light green) per cell type.

Since the CAG promoter is ubiquitous, we attempted to evaluate whether the transgene expression was detected in different cell types (i.e., neuronal and glial cells). Immunofluorescence double-staining analyses were performed at the striatum level, where the transduction efficiency was one of the strongest. In the putamen, in both AAV-PHP. B (Figure 4A) and AAV-PHP. eB (Figure 4B) conditions, double immunolabeling with the pan-neuronal marker NeuN or the well-established oligodendrocyte marker CNPase showed that EGFP was expressed predominantly in neurons and oligodendrocytes, suggesting a neuronal tropism. Scarce co-labeling between EGFP and glial fibrillary acidic protein (GFAP; astrocyte-specific cellular marker) or with the microglia-specific cellular marker, ionized calcium-binding adapter molecule 1(Iba-1) was also observed (Figures 4A,B). A quantitative unbiased counting evaluated the total number of EGFP-transduced cells and EGFP-negative cells (Table 2). For both variants, we confirmed that 80–90% EGFP-positive cells were approximately co-labeled with NeuN, 12–13% with Iba1, 10–15% with GFAP, and 80–90% with CNPase in the putamen (Figures 4A,B, bottom).

**TABLE 2:** Quantification of putaminal and hippocampal cell type distribution.

	Putamen						Hippocampus					
	AAV.PHP.B			AAV.PHP.eB			AAV.PHP.B			AAV.PHP.eB		
	GFP −	GFP+	Total	GFP −	GFP+	Total	GFP −	GFP+	Total	GFP −	GFP+	Total
NeuN	21	98	119	9	83	92	12	104	116	14	110	124
Iba1	39	6	45	29	4	33	26	10	36	28	15	43
GFAP	30	3	33	40	7	47	33	51	84	36	47	83
CNPase	21	90	111	6	95	101	20	12	32	27	44	71

### Both Vectors Preferentially Targets Neurons in the Hippocampus

**FIGURE 5:**
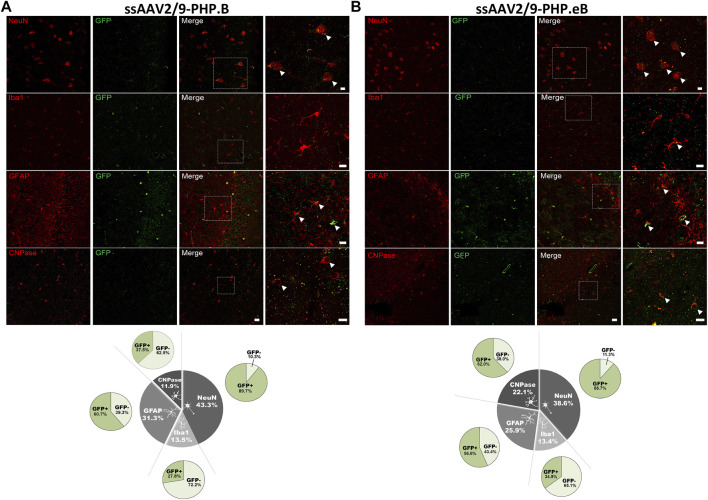
Assessment of the cell-type specificity resulting from AAV-PHP. B and AAV-PHP. eB injections in the hippocampus of non-human primates. **(A–B)** Representative images (up) and quantifications (down) of striatal sections from AAV-PHP. B-injected monkey **(A)** and AAV-PHP. eB-injected monkey **(B)** immunofluorescently labeled with anti-GFP antibody (green) and either a neuronal (NeuN), microglia (Iba1), astrocytic (GFAP + S-100) or oligodendroglial (CNPase) marker (red). Merging of the signals produced colocalization, thus confirming cellular transduction. Scale bar: 20 µm; 10 µm (high magnification). White arrows represent the AAV-GFP transduced cells. Quantitative analysis of percentage of cell types counted for each viral vector in the hippocampus of injected monkeys (grey) and percentage of cells transduced (dark green) or not (light green) per cell type.

To assess whether the neuronal and oligodendroglial tropism observed in the putamen was also identical in other brain regions with a different distribution of cell types, we investigated the cellular transgene expression of EGFP in the hippocampus of both the AAV-PHP. B (Figure 5A) and AAV-PHP. eB (Figure 5B) condition (Table 2). While the neuronal tropism remained sustained for both variants in the hippocampus (with 89–90% of EGFP-positive cells that were co-labeled with NeuN), we observed a higher astrocytic transduction for both variants (with 57–61% of EGFP-positive cells that were co-labeled with GFAP/S100) and a lower oligodendroglial transduction, especially for the AAV-PHP. B variant (with 38–62% of EGFP-positive cells that were co-labeled with CNPase) in the hippocampus compared to the putamen (Figures 5A,B). These data demonstrate that intrathecal delivery of both AAV-PHP. B and AAV-PHP. eB leads predominantly to neuronal transduction and is region-specific for oligodendrocyte and glial tropism.

### Both Vectors Achieved Weak GFP Transduction in the Spinal Cord and Liver

**FIGURE 6:**
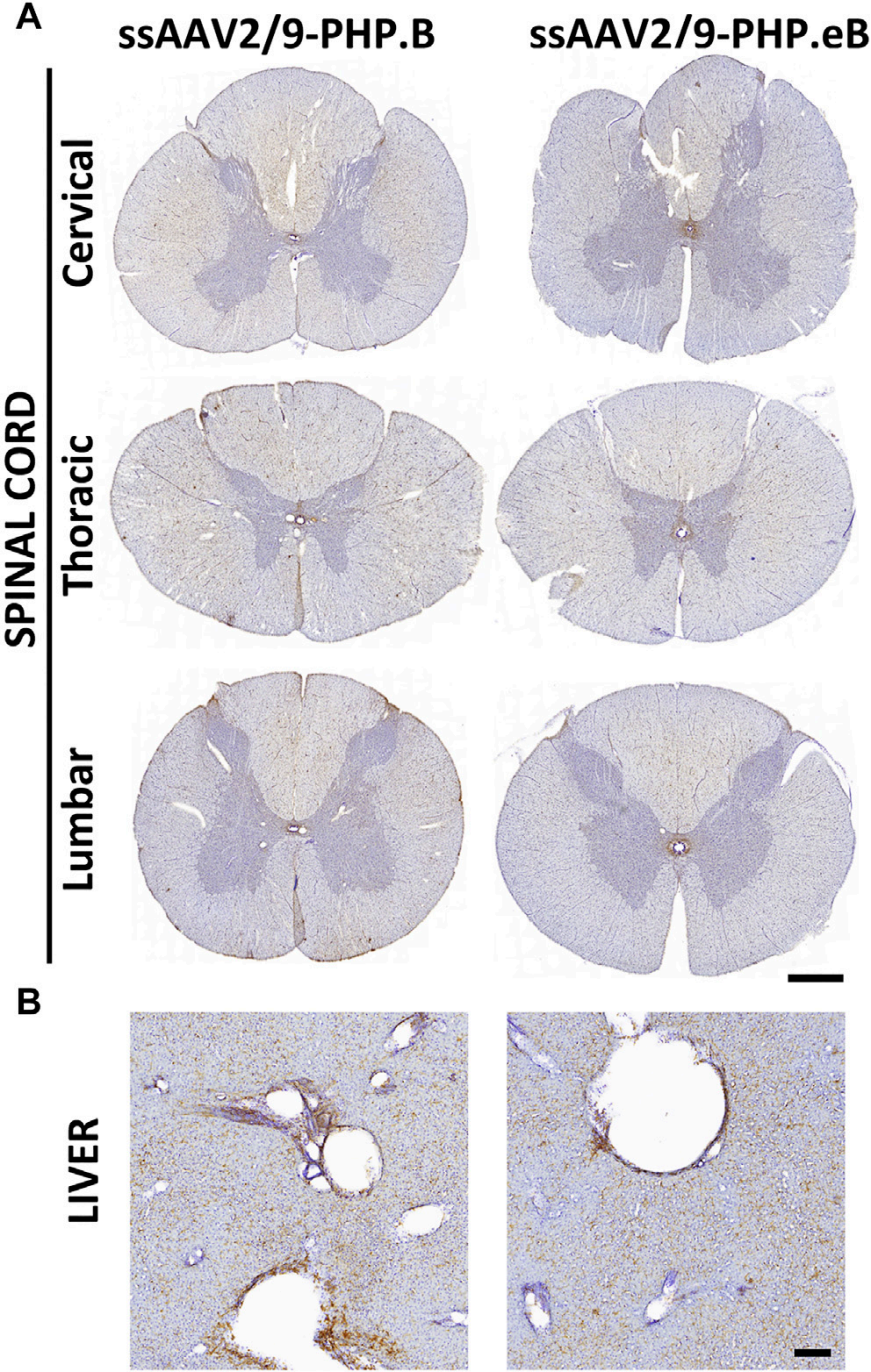
Broad transduction of the spinal cord and liver resulting from AAV-PHP. B and AAV-PHP. eB injections in non-human primates. **(A)** Representative image of spinal cord sections at three levels (cervical, thoracic, lumbar) immunostained with EGFP in both injected monkeys. Scale bar: 1 mm. **(B)** Representative image of liver sections immunostained with a GFP antibody in both injected monkeys. Scale bar: 200 µm.

While intrathecal delivery leads to convincing forebrain transduction, EGFP-immunopositive cells and fibers were weakly labeled in the cervical, thoracic, and lumbar spinal cord segments in both AAV-PHP. B and AAV-PHP. eB conditions (Figure 6A). As expected with an intrathecal delivery, weak EGFP-positive expression was detected in the liver regardless of the virus subtype (Figure 6B), suggesting that transduction remained primarily limited to CNS.

## DISCUSSION

To date, no studies have tested AAV-PHP. eB alone and compared AAV-PHP. B *vs*. AAV-PHP. eB variants in NHPs. This pilot study evaluated an unmet face-to-face evaluation of a single administration of equal doses of AAV-PHP. B and AAV-PHP. eB (1.5 × 10^11^ vg/ kg) to determine the potential use of those AAV9-derived variants in NHPs to transduce neural cells throughout the CNS, as successfully observed in rodents (Bedbrook et al., 2018). We decided to select one scalable and relatively noninvasive route of administration. Several reports have shown that intrathecal delivery of AAV vectors can enhance CNS penetration and optimize the ratio of CNS versus peripheral transduction (Gray et al., 2013; Castle et al., 2018; Hinderer et al., 2018; Liguore et al., 2019; Mathiesen et al., 2020). This minimally invasive intrathecal gene delivery system has the advantage of avoiding the occurrence of inflammation in the region surrounding the injection site during intracerebral injection. Moreover, intrathecal delivery of AAV2/9-gene therapies has been employed in clinical trials to treat giant axonal neuropathy (Bailey et al., 2018) and spinal muscular atrophy (Federici et al., 2012; Passini et al., 2014). Using a CAG promoter, which is ubiquitous and strong, to drive EGFP expression in both vectors, we observed widespread CNS transduction throughout the brain of both AAV-PHP. B- and AAV-PHP. eB-infused animals. Our study highlighted the superiority of AAV-PHP. eB CNS tropism and a more intense expression of EGFP than AAV-PHP. B in rhesus macaques. In addition, similar cell population-specific targeting was observed for both variants, mainly represented in neurons in the putamen and the hippocampus, while the transduction in non-neuronal cells seems to be more region-specific. Recently, (Matsuzaki et al., 2018) reported the lack of CNS tropism of AAV-PHP. B in the marmoset monkeys after i.v. administration at a dose of 5.0 × 10^13^ vg/ kg, questioning either the choice of the route of administration or differences between NHP species regarding the AAV transduction efficiency. In addition, (Hordeaux et al., 2018) reported that AAV-PHP. B did not enhance transduction in the rhesus macaque brain following i.v. injection. On the contrary, we reported here the transduction of neurons in the brain of the rhesus macaques, even at a 130-fold lower dose than the dose used by the authors (1.5 × 10^11^ vg/ kg compared with 2.0 × 10^13^ vg/ kg). Lately, (Liguore et al., 2019) evaluated transduction efficiency of AAV-PHP. B (but not AAV-PHP.eB) in the rhesus macaque following four distinct injection strategies. Following intrathecal administration at a dose of 1.0 × 10^12^ vg/kg, they observed broad cortical and spinal cord transduction, supporting our findings. Recently, (Galvan et al., 2021) demonstrated that a single intracerebroventricular injection of AAV9-PHP.B in two adult rhesus macaques (1.87 × 10^12^ vg/ kg and 3.95 × 10^12^ vg/ kg)., using a gene promoter that confers high neuron-specific expression of the transgene, the human synapsin 1 (SYN1), to drive the expression of emerald green fluorescent protein (EmGFP) in neurons, was efficient to induce widespread transgene expression in various populations of cortical and subcortical neurons in the primate brain.

While this first data set is encouraging, pending questions await future investigation before translating our findings to eventual use in gene therapy applications. We believe that it is crucial to accomplish several additional experiments on NHPs to lay a critical ground for a more systematic investigation in the CNS, peripheral (PNS), and enteric (ENS) nervous systems. Careful studies are needed to determine the precise pathological and molecular mechanisms involved and the potential implication for clinical trials using comparable doses of systemic vectors. Further investigations with those AAV9 capsid variants, particularly with the less characterized AAV-PHP. eB, should be conducted with additional animals to reach statistical power. Towards this goal, we propose different aspects of refinement that can be studied in a full-scale study in the near future. First, age should be considered as a variable (juvenile *vs*. mature), and inherent differences might exist in how AAV-PHP. B and AAV-PHP. eB transduce rhesus macaque brain. Second, single versus multiple injections may allow improving biodistribution throughout the brain. Third, the dose may be a crucial parameter, as in one NHP study, using AAV-PHP. B at a dose of 7.5 × 10^13^ vg/ kg led to unexpected AAV-mediated toxicity after a single i.v. injection (Hordeaux et al., 2018). Fourth, the specificity of expression being driven by structure or cellular-specific promoters within the vector genome should also be considered a way to control what cell types and under what conditions the transgene is expressed (Smith et al., 2021).

Altogether, this proof-of-concept experiment demonstrates that AAV-PHP. B and AAV-PHP. eB are robust viral vectors for gene transfer, notably to the brain, providing a promising research tool for delivering genes to NHPs. The present data have broad implications in brain diseases and constitute a dramatic step toward using engineered AAV9 capsid variants vectors to model human disease onset and deliver gene therapies to the CNS in humans. Interestingly, in the field of synucleinopathies, where neurons and oligodendrocytes are the primary cells involved in Parkinson’s disease and multiple system atrophy, respectively, the use of those viral vectors may be an attractive approach to target multiple affected brain cells. Regarding the field of tauopathy, the neuronal and oligodendroglial tropism of these vectors would be of interest to model four-repeat (4R-) tauopathies, for instance (progressive supranuclear palsy (PSP) and corticobasal degeneration (CBD), where neuronal inclusions and coiled bodies are hallmarks of the disease (Rosler et al., 2019).

## Data Availability

The raw data supporting the conclusions of this article will be made available by the authors, without undue reservation.
